# Directionality of substrate translocation of the hemolysin A Type I secretion system

**DOI:** 10.1038/srep12470

**Published:** 2015-07-27

**Authors:** Michael H. H. Lenders, Stefanie Weidtkamp-Peters, Diana Kleinschrodt, Karl-Erich Jaeger, Sander H. J. Smits, Lutz Schmitt

**Affiliations:** 1 Institute of Biochemistry, Heinrich-Heine-Universitaet, D-40225 Duesseldorf, Germany; 2 Center for Advanced Imaging (CAi), Heinrich-Heine-Universitaet, D-40225 Duesseldorf, Germany; 3 Protein Production Facility, Heinrich-Heine-Universitaet, 40225 Duesseldorf, Germany; 4 Institute of Molecular Enzyme Technology, Heinrich-Heine-Universitaet and Institute of Bio- and Geosciences IBG-1: Biotechnology, Forschungszentrum Juelich GmbH, D-52426 Juelich, Germany; 5 Center of Excellence on Plant Sciences (CEPLAS), Heinrich-Heine-Universitaet, D-40225 Duesseldorf, Germany

## Abstract

Type 1 secretion systems (T1SS) of Gram-negative bacteria are responsible for the secretion of various proteases, lipases, S-layer proteins or toxins into the extracellular space. The paradigm of these systems is the hemolysin A (HlyA) T1SS of *Escherichia coli*. This multiple membrane protein complex is able to secrete the toxin HlyA in one step across both *E. coli* membranes. Common to all secreted T1SS substrates is a C-terminal secretion sequence being necessary as well as sufficient for secretion. However, it is not known whether transport occurs directionally, i.e. the N- or the C-terminus of T1SS substrates is secreted first. We have addressed this question by constructing HlyA fusions with the rapidly folding eGFP resulting in a stalled T1SS. Differential labeling and subsequent fluorescence microscopic detection of C- and N-terminal parts of the fusions allowed us to demonstrate vectorial transport of HlyA through the T1SS with the C-terminus appearing first outside the bacterial cells.

Gram-negative bacteria transport a broad range of compounds ranging from small molecules to intact proteins into the extracellular space. Transport occurs *via* dedicated cellular nanomachineries either directly from the cytoplasm to the extracellular space or *via* periplasmic intermediates. Among these nanomachineries, type 1 secretion systems (T1SS) adopt the most simple architecture consisting of an ATP-binding cassette (ABC) transporter and a membrane fusion protein (MFP) located in the inner membrane and an outer membrane protein (OMP). In the presence of the substrate, they form a continuous tripartite channel reaching directly from the cytoplasm into the extracellular space^1^,^2^. The secretion of substrates occurs in one step across both, the inner and outer membrane of Gram-negative bacteria, without a periplasmic intermediate.

T1SS substrates include adenylate cyclases, lipases, proteases, surface layer proteins and toxins. They vary in size from relatively small proteins such as the hemophore HasA (19 kDa, 188 amino acids) from *S. marescens* to large proteins of approximately 900 kDa (8682 amino acids) such as the adhesion factor LapA from *P. fluorescence*^3^,^4^,^5^. These secreted proteins have in common that the information for secretion is encoded within the 50–60 C-terminal amino acids that were shown to be essential and sufficient for the secretion process^6^,^7^,^8^,^9^,^10^. This is in contrast to other protein translocation systems localized in the inner membrane such as the Sec system where the secretion signal is localized at the N-terminus^11^ and cleaved during translocation by a dedicated peptidase.

The most prominent substrates of T1SS are the repeats in toxin (RTX) proteins (Supplementary Fig. 1). They are characterized by glycine-rich repeats (GG repeats) located in the C-terminal part of the respective protein upstream of the secretion signal. The consensus sequence of the GG repeats forming the RTX domain^12^ of these proteins is GGxGxDxUx, where x can be any amino acid and U is a large or hydrophobic amino acid^13^. The number of these repeats depends on the size of the secreted protein and they are in general separated by number of amino acids, totaling on average 6–12 kDa within the RTX domain^13^,^14^. The GG repeats bind calcium thereby promoting folding of the secreted substrate^15^. Since the level of calcium is low (approximately 300 nM) in the cytosol of *E. coli*, but high (up to 10 mM) in the extracellular space^16^, it is generally assumed that T1SS substrates adopt their final, folded conformation only after secretion into the extracellular space^16^.

One of the best-characterized T1SS substrates is the pore forming RTX toxin hemolysin A (HlyA). HlyA is an alpha toxin, consisting of 1024 amino acids with a molecular weight of 110 kDa. Six consensus GG repeats are present within the RTX domain of HlyA^14^. The HlyA specific T1SS consists of the ABC transporter hemolysin B (HlyB), the MFP hemolysin D (HlyD) and the endogenously expressed OMP, TolC. HlyA is transported in one step from the cytoplasm to the extracellular space^2^. It has been demonstrated that the secretion signal of HlyA initiates secretion and is responsible for the assembly of the T1SS complex. HlyA is secreted in an unfolded state^17^ and its secretion signal contains all information necessary for secretion since it can be secreted alone^7^. High levels of secretion were also obtained with a fragment consisting of the 218 C-terminal amino acids of HlyA (HlyAc) containing three GG repeats and the secretion signal^17^.

Importantly, the orientation of the substrate during secretion is currently not known. The question arises whether directionality of secretion exists and if so, whether the N- or C-terminus of HlyA is translocated first through the T1SS. In general, at least two possibilities exist for the passage of the substrate through a T1SS. Since HlyB interacts with the secretion signal of HlyA with its nucleotide-binding domain^18^, one could envision that such binding to the nucleotide-binding domains of HlyB or to the cytoplasmic part of HlyD^19^ would stabilize the C-terminal part of the HlyA in the vicinity of the translocon. Furthermore, the so-called C39 peptidase -like domain (CLD) at the N-terminus of HlyB interacts with the substrate *via* binding of the RTX region of HlyA to facilitate secretion in some way^20^. This interaction might stabilize the interaction of the inner membrane components of the T1SS with the RTX domain and the secretion sequence providing further support for the idea that the N-terminal part enters the translocation channel first, with the final release of the C-terminal of HlyA into the translocon to complete translocation.

On the other hand C-terminal directed secretion might also be envisaged since many heterologous passenger proteins fused at their C-terminus to a C-terminal fragment of HlyA (containing the secretion signal) are secreted^17^,^21^,^22^,^23^,^24^. Intuitively, one would expect that the secretion process can only start after translation of the secretion signal. Since all fusion proteins have only the C-terminal secretion signal in common, one could envisage that the C-terminal part of these fusion proteins is secreted first. However, these two alternatives have not been addressed experimentally so far.

Here, we describe that a fusion of the enhanced Green Fluorescence Protein (eGFP) to the N-terminus of HlyAc stalled in the HlyA T1SS, presumably due to the fast folding properties of eGFP with a refolding half-time of 90.6 s^25^. Apparently, the eGFP-HlyAc fusion protein is fixed and stably oriented within the translocator *in vivo*. We only observed the N-terminal fragment within the cytoplasm whereas the C-terminal fragment was exclusively detected at the cell surface. Our results clearly demonstrate the presence of directionality during secretion with the C-terminal secretion sequence transported first. Our data further suggest that the secretion sequence is responsible for inserting the substrate into the T1SS.

## Results

### Stalling the T1SS with fast folding proteins

The secretion signal of HlyA is essential and sufficient for secretion of HlyA by its cognate T1SS^22^,^26^,^27^. HlyAc, a truncated version of HlyA containing the 218 C-terminal amino acids including the secretion signal, is secreted in high amounts comparable to full-length HlyA when expressed simultaneously with the inner membrane components HlyB and HlyD^17^,^23^,^24^,^28^. Interestingly, if HlyAc is fused to the C-terminus of, for example, the maltose binding protein (MBP), secretion is completely abolished. However, secretion can be restored with MBP fusion proteins that contain mutations reducing the folding rates^17^. Similar behavior has been observed for the Has secretion system where only unfolded HasA could be exported by its cognate ABC transporter, whereas the presence of folded cytosolic HasA resulted in an inhibition of secretion of its unfolded isoform^29^. This led to the conclusion that T1SS substrates, including HlyA, are transported in an unfolded state. This assumption is in line with the fact that calcium ions are required for folding of HlyA, which is prevented in the cytoplasm due to the low concentration of calcium ions^16^,^30^,^31^. These data suggest that HlyAc fusion to a fast folding passenger could block the translocator resulting in a stalled T1SS. If so this should allow us to address the question whether, with such a stalled intermediate, the secretion signal localized inside the cytosol, inside the translocation machinery or on the extracellular side.

In order to confirm that such a stalled intermediate does indeed form, we performed a competition experiment, in which HlyAc as well as an eGFP-HlyAc fusion were expressed in combination with the inner membrane components of the T1SS. Genes encoding both proteins, HlyAc and eGFP-HlyAc, were present on the same plasmid, but their expression could be induced independently of each other (further details are provided in material and methods).

**Figure 1:**
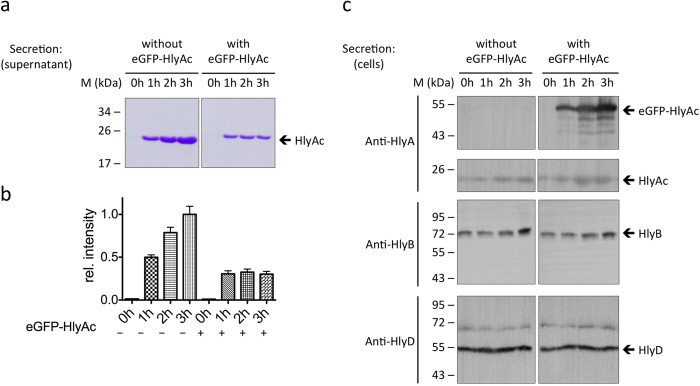
The eGFP-HlyAc fusion protein stalls the translocator and prevents HlyAc secretion. (**a**) SDS-PAGE analysis of the HlyAc secretion level (with Coomassie blue staining) in the culture supernatant over time without and with induction of eGFP-HlyAc expression. (**b**) Relative intensity of the SDS-PAGE bands illustrates HlyAc secretion levels without and with induction of eGFP-HlyAc. (**c**) Western blot analysis of the cells (comparison with the supernatants in (**a**)) show that eGFP-HlyAc is only present in the induced cells and that the levels of HlyB and HlyD remain constant over time.

Upon expression of the T1SS and both proteins, HlyAc and eGFP-HlyAc, a drastic reduction of the secretion levels of HlyAc was observed already after one hour of expression (Fig. 1a), clearly indicating competitive inhibition of HlyAc by the fusion protein. Quantification of the Coomassie stained protein bands normalized to the signal with the highest intensity, suggested that the amount of HlyAc in the supernatant was reduced to 30% when eGFP-HlyAc was co-expressed (Fig. 1b). The amount of secreted HlyAc in the presence of eGFP-HlyAc remained constant over time (Fig. 1b). This strongly suggests competition between the expressed HlyAc and eGFP-HlyAc consistent with stalling of the translocon by the latter already within the first hour, leading to substantially reduced amounts of secreted HlyAc.

The presence of intracellular eGFP-HlyAc was confirmed by Western blot analysis with a polyclonal HlyA antibody (Fig. 1c). The data show that the amount of cytosolic eGFP-HlyAc increased throughout incubation following induction of expression and furthermore, HlyAc was also detected within the cells. Thus, a direct comparison of the amounts of HlyAc and eGFP-HlyAc is possible. In cells expressing only the T1SS and HlyAc, the amount of cytosolic HlyAc remained constant over time whereas HlyAc accumulated in cells co-expressing eGFP-HlyAc and HlyAc (Fig. 1c). This observation indicates that eGFP-HlyAc has stalled the T1SS and secretion of HlyAc is blocked.

Western blot analysis confirmed as found in all subsequent experiments that the expression levels of HlyB and HlyD remained similar throughout incubation (Fig. 1c) and were not affected by expression of proteins to be secreted.

### T1SS is stalled only in the presence of the secretion signal

**Figure 2:**
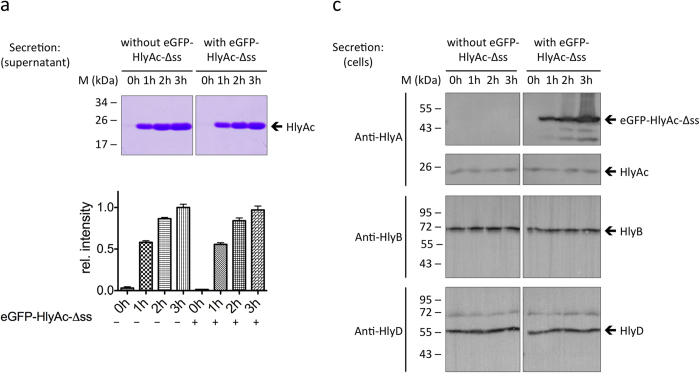
The eGFP-HlyAc-Δss fusion protein is unable to block the translocator. (**a**) SDS-PAGE analysis of the HlyAc secretion level in the culture supernatant over time without and with induction of eGFP-HlyAc-Δss expression. (**b**) Relative intensity of the SDS-PAGE bands illustrates the HlyAc secretion levels without and with induction of eGFP-HlyAc-Δss. (**c**) Western blot analysis of the cells (correlating to the supernatants in (**a**)) show that eGFP-HlyAc-Δss is only present in the induced cells and that the level of HlyB and HlyD remain constant over time.

Previous studies showed that the C-terminal located secretion signal of HlyA is crucial and necessary for secretion^23^,^32^. To analyze its role for stalling the secretion machinery, we engineered an eGFP-HlyAc fusion protein lacking the secretion signal (i.e. with the C-terminal 60 residues deleted) designated eGFP-HlyAc-Δss (Supplementary Fig. 4). A competition experiment (Fig. 2a) with independently induced expression of HlyAc and eGFP-HlyAc-Δss revealed that in the presence of HlyB and HlyD, now an inhibition of secretion of HlyA, did not occur. In contrast to the results shown in Fig. 1, the co-expression of HlyAc and eGFP-HlyAc-Δss in the presence of HlyB and HlyD did not result in a detectable reduction of the secretion levels of HlyAc with equal amounts of secreted HlyAc after three hours in the two experimental set-ups (Fig. 2b). Western blot analysis confirmed that the T1SS target protein eGFP-HlyAc-Δss as well as the corresponding transporter proteins HlyB and HlyD were expressed at a similar level under both experimental conditions (Fig. 2c).

### The HlyAc fragment of the fusion protein is exposed on the cell surface accessible to HlyA antibody

The secretion signals of HlyA as well as of other T1SS substrates are localized at the extreme C-terminus. The competition experiments described above demonstrated that the HlyA secretion signal is also required for stalling the secretion machinery. To address the question, whether the C- or the N-terminal of the fusion appears first on the surface, we took advantage of the eGFP fluorescence (Supplementary Fig. 5). This fluorescence allowed the detection of the eGFP part of the fusion protein, while the HlyAc part can be visualized *via* an HlyA specific antibody in combination with a secondary antibody that harbors the fluorophore Cy3 resulting in a red fluorescence.

If the N-terminus is transported first, intrinsic eGFP fluorescence but no red fluorescence should be detectable extracellularly because the Cy3-labeled second antibody can only bind to the HlyA specific first antibody if part of HlyAc has exited the TolC component of the translocon to the exterior. In contrast, if the C-terminal part of the fusion protein is transported first, one would detect red fluorescence in combination with the green fluorescence derived from intracellular eGFP.

**Figure 3:**
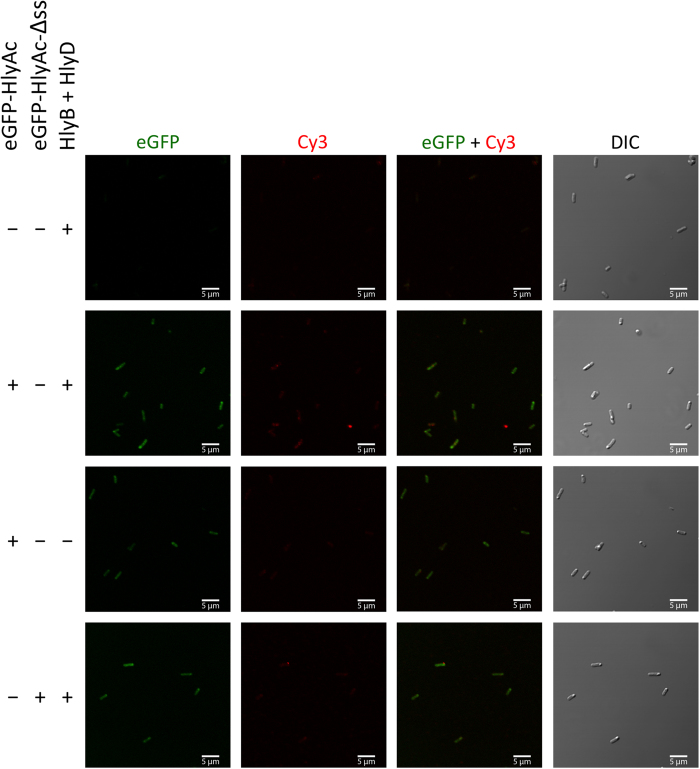
Detection of the surface exposed HlyAc fragment of eGFP-HlyAc by confocal laser scanning microscopy. *E. coli* cells expressed HlyB and HlyD, as well as additional eGFP-HlyAc and eGFP-HlyAc-Δss. Shown is the eGFP fluorescence (left panel) of the fusion proteins, the HlyA mediated Cy3 fluorescence at the cell surface (second left panel), merged images of eGFP and Cy3 fluorescence (second right panel) and differential interference contrast (DIC) images of the cells (right panel). The different combinations of proteins employed are indicated to the left.

*E. coli* cells containing a stalled T1SS were analyzed with confocal laser scanning microscopy (CLSM). Cells expressing only HlyB and HlyD, but lacking the eGFP-HlyAc encoding plasmid were used to determine the cellular autofluorescence (Fig. 3, top row). The expression of HlyB and HlyD was confirmed by Western blots analysis (Supplementary Fig. 6).

Next, we analyzed cells producing HlyB, HlyD and eGFP-HlyAc. CLSM images confirmed that both eGFP as well as Cy3 fluorescence was detected (Fig. 3, second row). The eGFP fluorescence signal was found homogenously distributed within the cells, although sometimes accumulating at the cell poles and the Cy3 fluorescence signal also appeared evenly distributed over the cells. Due to the limited optical resolution of CLSM, the localization of both proteins could not be analyzed more precisely.

**Figure 4:**
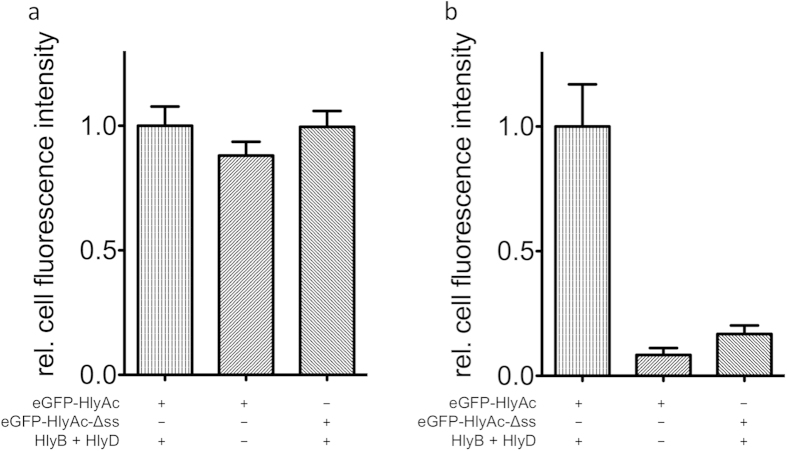
(**a**) Relative cell fluorescence of eGFP. All values were normalized to the eGFP fluorescence of the eGFP-HlyAc fusion protein (error bars represent the standard error of the mean) after subtraction of autofluorescence. (**b**) Relative fluorescence of Cy3. All values are normalized to Cy3 fluorescence of the eGFP-HlyAc fusion protein (error bars represent the standard error of the mean) after subtraction of autofluorescence. The different combinations of proteins employed are indicated below the bars.

The eGFP fluorescence in cells expressing the different constructs was quantified by normalization of eGFP intensity values to the fluorescence intensities measured with cells expressing HlyB, HlyD and eGFP-HlyAc (Fig. 4a, left bar).

The Cy3 fluorescence of cells expressing HlyB, HlyD and eGFP-HlyAc reflected the presence of HlyA antibodies bound to the cell surface and Cy3 fluorescence intensity values were normalized to this value (Fig. 4b, left bar).

As a control, cells expressing eGFP-HlyAc, but not HlyB and HlyD, were also analyzed. The CLSM images confirmed that eGFP fluorescence could be detected in the cytosol of cells (Fig. 3, third row). Quantification of the eGFP and Cy3 fluorescence demonstrated that eGFP fluorescence was approximately 88 ± 5.5% as compared to cells expressing the eGFP-HlyAc fusion protein (Fig. 4a, middle bar) whereas almost no Cy3 fluorescence could be detected (8 ± 2.8%, Fig. 4b, middle bar).

The competitive secretion assay with eGFP-HlyAc-Δss revealed that the secretion signal was needed for stalling of the translocator. Accordingly, cells expressing eGFP-HlyAc-Δss, HlyB and HlyD were analyzed by CLSM confirming that eGFP fluorescence was detected in the cytosol (Fig. 3, fourth row). eGFP fluorescence is approximately 100 ± 6.3% compared to cells expressing the eGFP-HlyAc fusion protein (Fig. 4a, right bar) and only 17 ± 3.4% of Cy3 fluorescence in comparison to cells expressing HlyB, HlyD and eGFP-HlyAc was detected (Fig. 4b, right bar).

In summary, these results demonstrate that the eGFP-HlyAc fusion protein is specifically orientated during secretion. The constructs used here allow the assignment of the N-terminal eGFP fluorescence to the cytosol, whereas the C-terminal part of the fusion protein harboring the HlyAc fragment is exposed at the cell surface and accessible to antibody.

### Analysis of the secretion of full-length eGFP-HlyA

Next, cells expressing HlyB, HlyD and a fusion protein of eGFP and full-length HlyA (eGFP-HlyA) were analyzed. Due to the fast folding of eGFP, the eGFP-HlyA fusion should not be secreted and should also stall the T1SS.

**Figure 5:**
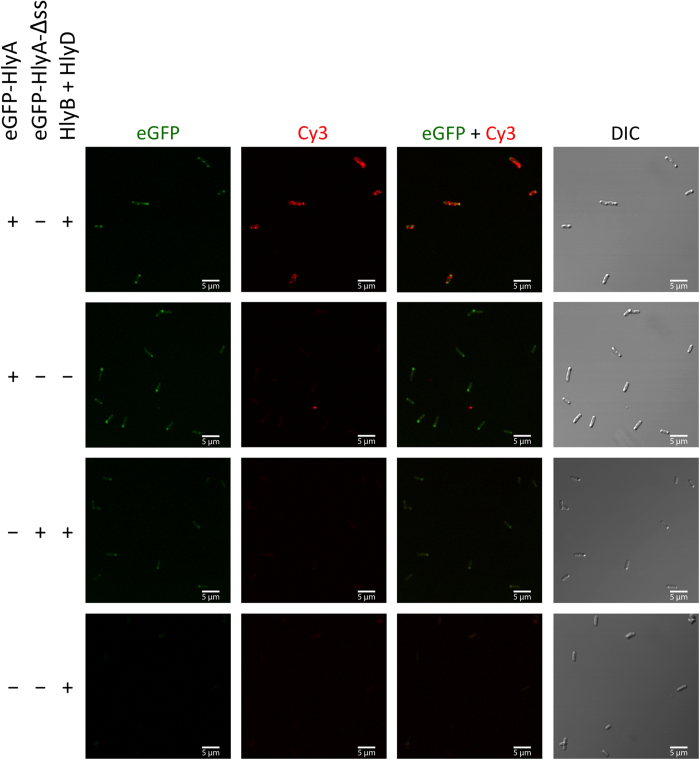
Detection of the surface exposed HlyA fragment of eGFP-HlyA by confocal laser scanning microscopy. *E. coli* cells expressed HlyB and HlyD, as well as additional eGFP-HlyAc and eGFP-HlyAc-Δss, respectively. Shown is the eGFP fluorescence (left panel) of the fusion proteins, the HlyA mediated Cy3 fluorescence at the cell surface (second left panel), merged images of eGFP and Cy3 fluorescence (second right panel) and differential interference contrast (DIC) images of the cells (right panel). The different combinations of proteins tested are indicated to the left.

**Figure 6:**
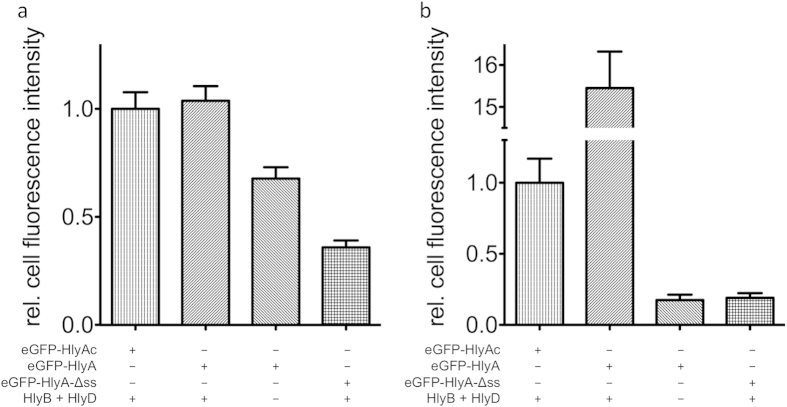
(**a**) Relative cell fluorescence of eGFP. All values were normalized to the eGFP fluorescence of the eGFP-HlyAc fusion protein (error bars represent the standard error of the mean) after subtraction of autofluorescence. (**b**) Relative fluorescence of Cy3. All values are normalized to Cy3 fluorescence of the eGFP-HlyAc fusion protein (error bars represent the standard error of the mean) after subtraction of autofluorescence. The different combinations of proteins employed are indicated below the bars.

CLSM images confirmed that eGFP and Cy3 fluorescence were observed (Fig. 5, first row). Quantification of the eGFP and Cy3 fluorescence intensities of the cells demonstrated that the eGFP fluorescence was approximately 103 ± 6.8% (Fig. 6a, second bar on the left) compared to cells that expressed the eGFP-HlyAc fusion protein (Fig. 6a, first bar from the left). Localization of eGFP fluorescence appeared identical to cells expressing the eGFP-HlyAc fusion together with HlyB and HlyD (Fig. 3, second row) confirming that the eGFP part of the fusion protein is localized in the cytoplasm. Cy3 fluorescence reflecting the binding of anti-HlyA antibody at the cell surface was ten times higher (Fig. 6b, second bar from the left) than Cy3 fluorescence of cells expressing eGFP-HlyAc (Fig. 6b, first bar from the left). Obviously, the full-length Hly A provides many more epitopes for binding of the polyclonal HlyA antibody than the HlyAc. Furthermore, staining revealed a heterogeneous distribution of HlyA protein showing accumulations on the cell surface. This result confirmed that the C-terminal portion of eGFP-HlyA is exposed on the cell surface.

These results clearly indicate that a fusion protein consisting of eGFP and full-length HlyA was recognized and transported by the T1SS. However, secretion was stalled with a high probability with eGFP detected in the cytoplasm as shown by the corresponding eGFP fluorescence signal. In control experiments either with cells not expressing the translocator components of the inner membrane (Fig. 5, second row) or eGFP-HlyA-Δss, HlyB and HlyD (Fig. 5, third row) greatly reduces levels of the C-terminal HlyA fragment were detected on the cell surface as indicated by specific Cy3 fluorescence resulting from binding of the HlyA antibody.

**Figure 7:**
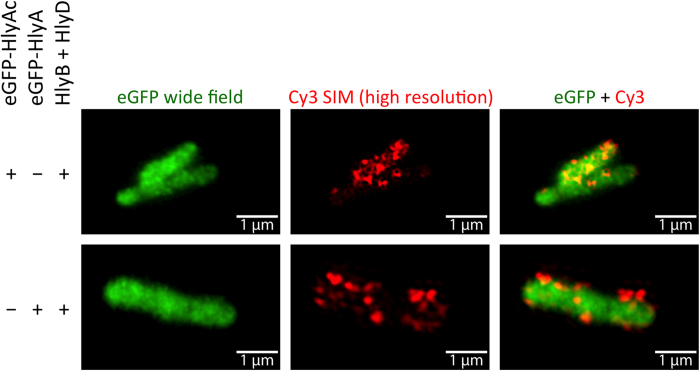
Detection of the surface exposed HlyA fragment of eGFP-HlyAc and eGFP-HlyA by structured illumination microscopy. Shown are maximum intensity projections of z-stacks of representative *E. coli* cells expressing eGFP-HlyAc or eGFP-HlyA together with HlyB and HlyD. The eGFP fluorescence (left panel in green) is displayed in wide field mode, the HlyA signal (medium panel in red) is displayed in high resolution mode after SIM processing. The right panel shows merged images derived from eGFP and Cy3 fluorescence recordings.

To gain more detailed insights into the cellular localization of the HlyA constructs we applied Structured illumination microscopy (SIM) to eGFP-HlyAc and eGFP-HlyA samples. To improve the optical resolution in SIM a grid pattern is repeatedly projected into the image plane in different orientations to produce interference patterns with sample structures. In a post-processing step a high-resolution image of the underlying structures can be generated using a computer algorithm. Under optimal conditions the lateral resolution in the resulting image is two times better than in a conventional confocal image^33^. As expected, Cy3 fluorescence was detected at the cell surface of cells expressing HlyB, HlyD and eGFP-HlyAc (Fig. 7, first row) and of cells expressing HlyB, HlyD and eGFP-HlyA (Fig. 7, second row) oriented in a distinct helical pattern. Also, wide field microscopy of eGFP fluorescence confirmed its cytosolic localization. Merged eGFP and Cy3 fluorescence signals further emphasized the extracellular localization of the Cy3 fluorescence, while the eGFP fluorescence was clearly localized in the cytoplasm (Fig. 7).

## Discussion

Recent experiments demonstrated that substrates of T1SS are secreted in an unfolded manner^17^. This observation is in line with geometrical restraints, since the interior diameter of TolC is maximally 20 Å^34^. Thus, assuming an average helical diameter of 12 Å, TolC could accommodate only a single α-helix. Consequently, substrates must enter the translocation machinery with their N- or C-terminus first, but a hairpin insertion is highly unlikely. Our experiments indeed demonstrated that fusion proteins of eGFP with HlyAc or HlyA stalled the T1SS, and furthermore, indicated that their C-terminal parts were exposed on the cell surface, while the N-terminal eGFP was always located in the cytosol in a folded state as deduced from its intrinsic fluorescence.

As show in the competition secretion assay the amount of HlyAc in the supernatant was reduced to 30% when eGFP-HlyAc was co-expressed (Fig. 2b). This suggests a competition between the expressed HlyAc and eGFP-HlyAc for free T1SS. In the first hour HlyAc is able to pass the cell membranes by the free T1SS and remain stable in the supernatant. After an hour the eGFP-HlyAc fusion protein blocks all T1SS and HlyAc is not able to secrete anymore. The level of HlyAc in the supernatant remains constant over the complete secretion time and contains only the stable HlyAc that was secreted in the beginning.

These results obviously raise the question whether the observed directionality of secretion represents a universal feature of T1SS. The inner membrane components of the ABC transporter can be divided in three different protein families defined by the presence or absence of additional domains. These families are ABC transporters with (I) C39 peptidase domains, (II) CLD domains, corresponding to an inactive peptidase which lack protease activity^20^ and (III) transporters without any additional domains^20^,^35^. Transporters with an authentic C39 peptidase domain transport rather small substrates (<10 kDa) that belong mainly to the bacteriocin family containing a cleavable N-terminal leader peptide for secretion (Supplementary Fig. 1). Due to the cleavable, N-terminal leader sequence and the fact that these systems are also present in Gram-positive bacteria, we shall exclude family (I) from our discussion.

Transporters with a CLD transport larger substrates (>55 kDa) that are all members of the RTX toxin family^20^,^35^. The RTX toxins have their secretion signals located at the C terminus (50–60 C-terminal amino acids) together with GG repeats located close to the secretion signal (Supplementary Fig. 1) that promote active folding of the secreted protein by binding of calcium ions in the extracellular medium^15^.

An example for transporters without any additional domains is the hemophore transporter HasD of *Serratia marcescens*^4^,^36^. Here, the C-terminal secretion signal is not cleaved during transport, but HasA, like other substrates of this family, does not contain conserved GG repeats (Supplementary Fig. 1). Instead, HasA appears to contain so-called primary recognition sites scattered throughout the protein and the secretion of HasA is dependent on SecB^36^,^37^; a feature that was not observed for substrates belonging to the RTX protein family^17^.

**Figure 8:**
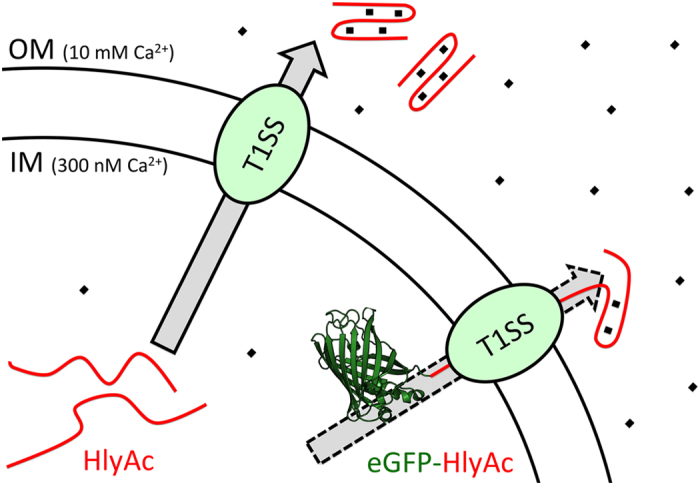
Model for secretion by the T1SS. The T1SS is indicated by an oval box and the substrate, here HlyA, is depicted as a red line, while eGFP is shown in green (pdb file 2Y0G). HlyAc is secreted (indicated by the grey arrow) and folds in the extracellular space due to the higher calcium concentration. eGFP-HlyAc can only enter the T1SS but is not able to complete the secretion process (indicated by the dotted grey arrow) due to the fast folding of eGFP and stalling inside the translocator. The model also assumes that cell surface exposed HlyAc fragment of the fusion protein folds due to the higher extracellular calcium concentration. Calcium ions are indicted by small black boxes. For further details see text.

From an energetic point of view, the initial appearance of GG repeats at the cell surface following translocation would allow binding of calcium ions thereby inducing folding of the secreted part of the protein. This process we propose would pull the protein through the T1SS^13^ and, in parallel, prevent back sliding into the secretion machinery because a tertiary structure would be formed which is larger in size than the maximal diameter of TolC (20 Å). This leads to the assumption that the C-terminus of RTX proteins may lead the way through the translocator and GG repeats appear at the cell surface thus providing the driving force for secretion (depicted schematically in Fig. 8). In line with these arguments is the observation that the amount of GG repeats correlates with the size of the secreted substrate^13^,^14^. Our results also suggest that the directionality of secretion determined for the HlyAc/HlyA fusion proteins is a universal feature of the RTX protein family, which may also apply for other T1SS substrates containing a C-terminal secretion sequence.

## Methods

### Bacterial strains and plasmids

The *E. coli* strain DH5α was used for all cloning procedures. The pK184 plasmid (Supplementary Fig. 2) was used for HlyB and HlyD production under the control of a P_*lac*_ promoter, inducible with IPTG (isopropyl β-D-1-thiogalactopyranoside)^17^. All plasmids and oligonucleotides used in this study are summarized in Supplementary Table 1 and 2.

Plasmid pSOI-eGFP-HlyAc was used for eGFP-HlyAc expression under the control of a P_BAD_ promoter. Plasmid pSOI-eGFP-HlyAc was cloned through the restriction free cloning (RF-cloning) method^38^. The *eGFP* gene was amplified by PCR using the vector pcDNA3-eGFP and primers RF_pSOI_eGFP_for and RF_pSOI_eGFP_rev to generate the mega-primer. Amplified eGFP gene and pSOI-HlyAc^17^ were used with a vector-to-insert-ratio of 1 to 2.5. After successful cloning, the eGFP-HlyAc-∆ss variant was created by using PCR where the base pairs encoding for amino acid position 425 to 485 were deleted. Primer pair Deletion-HlyAc-for and Deletion-HlyAc-rev was used.

The HlyAc gene was exchanged for full-length *hlyA* to generate plasmid pSOI-eGFP-HlyA by RF-cloning. The *hlyA* gene was amplified by PCR using plasmid pSU-*hlyA*^39^ and primers RF_pSOI_HlyA_for and RF_pSOI_HlyA_rev. The amplified genes were used with a vector-to-insert-ratio of 1 to 10. To generate plasmid pSOI-eGFP-HlyA-Δss, which expresses the eGFP-HlyA-∆ss variant lacking the secretion signal, a stop-codon was introduced by site directed mutagenesis applying the primers HlyAΔss_for and HlyAΔSS_rev.

For the simultaneous expression of eGFP-HlyAc and HlyAc, we constructed a plasmid (pSOI-eGFP-HlyAc^BAD^/HlyAc^lac^) designed for a co-expression of both target genes under the control of two different promoters (Supplementary Fig. 3). In the case of eGFP-HlyAc we chose a P_BAD_ promoter, while a P_*lac*_ promoter was selected for the expression of HlyAc. Cloning was achieved with the In-Fusion® Advantage PCR Cloning Kit (ClonTech). Therefore, plasmid pSOI-eGFP-HlyAc was linearized by PCR using primers pSOI_ColE1_for and pSOI-AMP_rev. The insert, the *hlyAc* gene with a P_*lac*_ promoter and terminator, was amplified by PCR applying pSU-*hlyA* as template with the primers Inf_pSOI_HlyA_F and Inf_pSOI_HlyA_R. The In-Fusion reaction with the linearized plasmid and the insert was performed according to the manufactures protocol. After successful cloning of pSOI-eGFP-HlyAc^BAD^/HlyAc^lac^, the eGFP-HlyAc-∆ss variant was created by deleting amino acids 425 to 485 as described above. The resulting plasmid pSOI-eGFP-HlyAc-Δss^BAD^/HlyAc^lac^ confers expression of HlyAc and the eGFP-HlyAc-∆ss fusion protein that lacks the secretion signal (Supplementary Fig. 4).

### Competition secretion assay

Chemically competent *E. coli* BL21 (DE3) cells were transformed with pK184-HlyBD and pSOI-eGFP-HlyAc^BAD^/HlyAc^lac^ or pSOI-eGFP-HlyAc-Δss^BAD^/HlyAc^lac^ and grown on LB agar plates supplemented with 100 μg mL^−1^ ampicillin and 30 μg mL^−1^ kanamycin. Overnight cultures of single colonies were used to inoculate 25 mL 2 YT medium supplemented with 100 μg mL^−1^ ampicillin and 30 μg mL^−1^ kanamycin at an OD_600_ of 0.1. Cultures were grown at 37 °C and 180 rpm. The expression of HlyAc, HlyB and HlyD was induced with 1 mM IPTG at an OD_600_ of 0.6–0.8. The secretion levels were significantly enhanced by addition of 5 mM CaCl_2_ (final concentration) to the culture media. Cells were grown for 3 h at 180 rpm and 37 °C to obtain sufficient amounts of the translocator components of the inner membrane and a constant expression and secretion level of HlyAc. Subsequently, cells were spun down for 15 min at 8000 g and re-suspended in fresh 2 YT media supplemented with 100 μg mL^−1^ ampicillin, 30 μg mL^−1^ kanamycin, 1 mM IPTG and 5 mM CaCl_2_. This procedure ensured removal of secreted HlyAc from the supernatant. To monitor newly secreted HlyAc in cells expressing the translocation machinery, HlyAc in combination with eGFP-HlyAc or eGFP-HlyAc-Δss, respectively, the culture was split into two halves. 10 mM arabinose was added to one culture for inducing expression of the fusion protein eGFP-HlyAc or eGFP-HlyAc-Δss. The culture without added arabinose was used as a control. Cells were grown for 3 h at 180 rpm and 37 °C. A 1 mL aliquot was taken and centrifuged for 5 min at 14000 g, 4 °C each hour during growth. Cells were adjusted with water to an OD_equivalent_ of 0.1 and supernatant samples were analyzed by SDS-PAGE and/or Western blot analysis.

The expression levels of HlyB and HlyD as well as the expression of eGFP-HlyAc or eGFP-HlyAc-Δss and the intracellular amount of HlyAc were determined via Western blots using polyclonal antibodies against HlyA, HlyB or HlyD in combination with an horseradish peroxidase (HRP)-conjugated, secondary antibody using the ECL advance kit (GE Healthcare).

### Cell cultivation and protein expression for confocal laser scanning microscopy

*E. coli* BL21 (DE3) were prepared and induced identically to cells used in the competition assay. Cells were grown for 2 h at 180 rpm and 37 °C and harvested by centrifugation and directly used for cell fixation and immunofluorescence labeling.

### Cell fixation by formaldehyde treatment

50 μL of cells grown until an OD_600_ of 2.0 were mixed with 3% (v/v) formaldehyde in 2 YT medium for fixation and incubated at 37 °C and 180 rpm for one hour.

### Immunofluorescence labeling of formaldehyde treated cells

Formaldehyde treated cells expressing eGFP-HlyAc, eGFP-HlyAc-Δss, eGFP-HlyA or pSOI-eGFP-HlyA-Δss in the presence or absence of HlyB and HlyD as well as cells that only expressed HlyB and HlyD were centrifuged at 11000 g for 5 min and re-suspended in the 50 μl PBS buffer containing 3% (w/v) BSA. Cells were transferred in a 1:50 diluted rabbit anti-HlyA antibody solution and incubated for one hour. Subsequently, cells were washed three times with PBS buffer containing 3% (w/v) BSA and suspended in a solution containing a secondary antibody conjugated with the fluorophore Cy3. Cells were incubated for one hour at 37 °C and washed two times in 50 μl PBS buffer.

### Confocal laser scanning microscopy and image processing

10 μL of labeled cells were mixed with 2 μL of ProLong®Gold antifade reagent on a poly-lysine coated slide, covered with a cover slip and sealed with clear varnish.

Imaging was performed with an Olympus FV1000 confocal laser scanning microscope (Olympus GmbH, Hamburg, Germany) equipped with a 60x oil immersion objective, numerical aperture 1.35. The eGFP fluorescence was excited at 488 nm using an argon laser at an output power of 5% and the Cy3 fluorescence was excited at 559 nm using a DPSS laser at 5%. The emission of eGFP was detected between 500 nm and 540 nm and the emission of Cy3 was detected between 565 nm and 600 nm using a spectral detector. Images of 1024 × 1024 pixels were taken with a pixel size of 0.05 μm.

The program ImageJ was used for processing and determination of cell fluorescence. The integrated intensity from all focused cells of all images of each type were determined and subtracted from the product of the mean background fluorescence multiplied by the area of the determined integrated intensity.

The program Prism 5.0 (GrapPad Inc) was used for a statistical analysis of the data. Mean cell fluorescence of each type of cell was determined together with the corresponding errors of the mean. Auto fluorescence represented by cells expressing only HlyB and HlyD were subtracted. Normalization was performed for eGFP and Cy3 fluorescence on the mean value of cells expressing eGFP-HlyAc, HlyB and HlyD (Figs 4 and 6).

### Structured illumination microscopy and image processing

Structured illumination microscopy (SIM) was performed on a Zeiss ELYRA PS.1 system (Carl Zeiss Microscopy GmbH, Goettingen, Germany) equipped with an Andor EM-CCD iXON DU-885 with 1004 × 1002 pixels. Z-stacks were taken using a 63x oil immersion objective with a numerical aperture of 1.46. To generate structured illumination a grid pattern was projected onto the image plane in five different positions and at five different modulation angles to obtain high frequency information within the low frequency information captured by the optical system. For the Cy3-channel back-computation of the lower frequencies using Fourier transformation was performed using the Zeiss ZEN Structured Illumination Processing tool to increase the resolution in the final image. Due to the almost homogeneous distribution of the eGFP signal there was no gain in resolution by image processing in the eGFP channel.

## Additional Information

**How to cite this article**: Lenders, M. H. H. *et al.* Directionality of substrate translocation of the hemolysin A Type I secretion system. *Sci. Rep.* **5**, 12470; doi: 10.1038/srep12470 (2015).

## Supplementary Material

Supplementary Information
