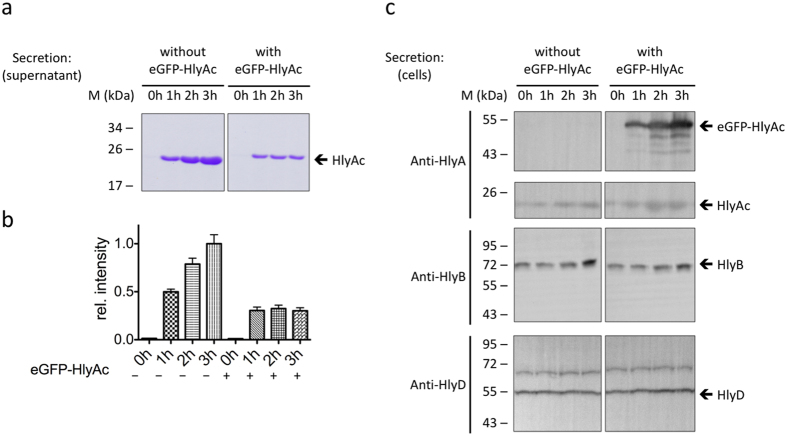# Corrigendum: Directionality of substrate translocation of the hemolysin A Type I secretion system

**DOI:** 10.1038/srep46926

**Published:** 2018-01-08

**Authors:** Michael H. H. Lenders, Stefanie Weidtkamp-Peters, Diana Kleinschrodt, Karl-Erich Jaeger, Sander H. J. Smits, Lutz Schmitt

Scientific Reports 5: Article number: 1247010.1038/srep12470; published online: 07 27 2015; updated: 01 08 2018

In this Article, figure 1 contains errors. Errors were made during the preparation of figure 1: the same HylD blot was accidentally used in figures 1 and 2. The correct Figure 1 appears below.

## Figures and Tables

**Figure 1:**