# Supplementary material for: Directionality of substrate translocation of the hemolysin A Type I secretion system
Source: Sci Rep. 2015 Jul 27;5:12470. doi: 10.1038/srep12470 (PMC4648471; doi:10.1038/srep12470)
Supplement: Supplementary Information [file srep12470-s1.docx]

Supplementary

**Directionality of substrate translocation of the hemolysin Type I secretion system**

Michael H. H. Lenders^1^, Stefanie Weidtkamp-Peters^2^, Diana Kleinschrodt^3^, Karl-Erich Jaeger^4,5^, Sander H. J. Smits^1^ and Lutz Schmitt^1,5^*

^1^Institute of Biochemistry, Heinrich-Heine-Universitaet, 40225 Duesseldorf, Germany

^2^Center for Advanced Imaging (CAi), Heinrich-Heine-Universitaet, 40225 Duesseldorf, Germany

^3^Protein Production Facility, Heinrich-Heine-Universitaet, 40225 Duesseldorf, Germany

^4^Institute for Molecular Enzyme Technology (IMET), Forschungszentrum Jülich, 52426 Jülich, Germany

^5^Center of Excellence on Plant Sciences (CEPLAS), Heinrich-Heine-Universitaet, 40225 Duesseldorf, Germany

*To whom correspondence should be addressed:

Lutz.Schmitt@hhu.de

Tel. +49 211 81-10773

Fax +49 211 81-15310

Universitaetsstraße 1

40225 Duesseldorf

Germany

Figures legends

Supplementary Fig. 1

Domain organization of different T1SS substrates. Boxes on the left highlight the ABC transporter families involved in the T1SS. “CLD” describes a T1SS with an ABC transporter with an N-terminal CLD extension, contributing defective peptidase, “C39” describes a T1SS with an ABC transporter having an active N-terminal C39 peptidase domain and “no” describes an ABC transporter without additional domains. Proteins are abbreviated as follows and listed with their corresponding UniProtKB entries: HlyA, hemolysin A; LktA, leukotoxin; RtxA, RtxA; CyaA, bifunctional hemolysin/adenylate cyclase; PaxA, exotoxin PaxA; CvaC, colicin V protein; ComC, competence-stimulating peptide type 1; HasA, hemophore HasA; EprA, metalloprotease EprA. Domains of the substrates are labeled as follows: AC, adenylate cyclase domain; RTX, RTX domain; GG, GG repeats; SEC, secretion signal; L, N-terminal leader peptide; MP, metalloprotease domain.

Supplementary Fig. 2

Plasmid map pK184-HlyBD. The map was created using the PlasMapper web server [^1^](#_ENREF_1).

Supplementary Fig. 3

Plasmid map pSOI-eGFP-HlyAc^BAD^ / HlyAc^lac^. The map was created using the PlasMapper web server [^1^](#_ENREF_1).

Supplementary Fig. 4

Plasmid map pSOI-eGFP-HlyAc-Δss^BAD^ / HlyAc^lac^. The map was created using the PlasMapper web server [^1^](#_ENREF_1).

Supplementary Fig. 5

Plasmid map pSOI-eGFP-HlyAc. The map was created using the PlasMapper web server [^1^](#_ENREF_1).

Supplementary Fig. 6

Western blot analysis of supernatants and total cells content of CLSM analyzed cells. eGFP-HlyAc respectively eGFP-HlyAc-Δss, HlyB and HlyD are only present if the corresponding promotors were induced.

Supplementary Fig. 7

Western blot analysis of supernatants and total cells content of CLSM analyzed cells. eGFP-HlyA respectively eGFP-HlyA-Δss, HlyB and HlyD are only present if the corresponding promotors were induced.

Figures


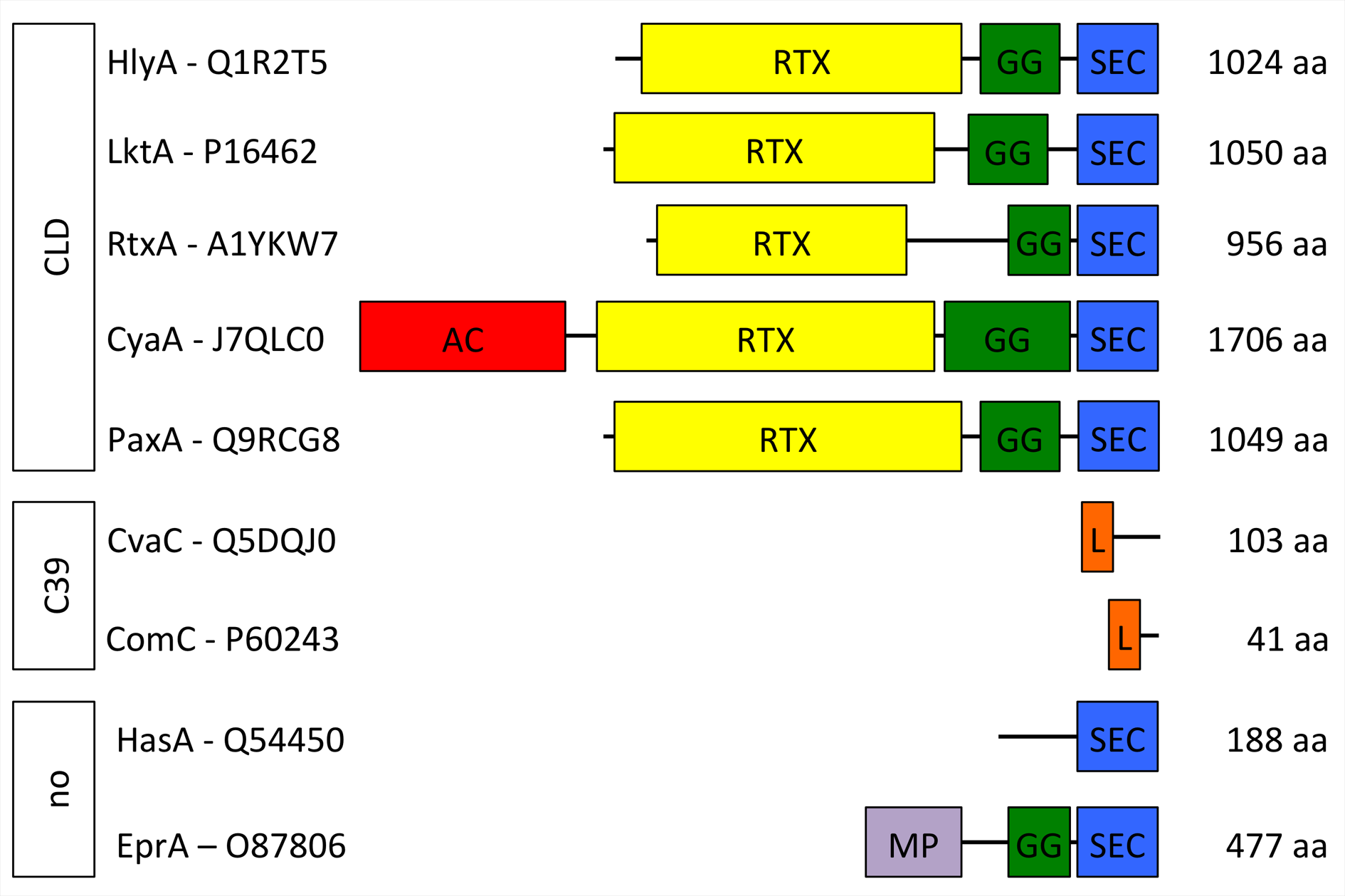


Supplementary Figure 1


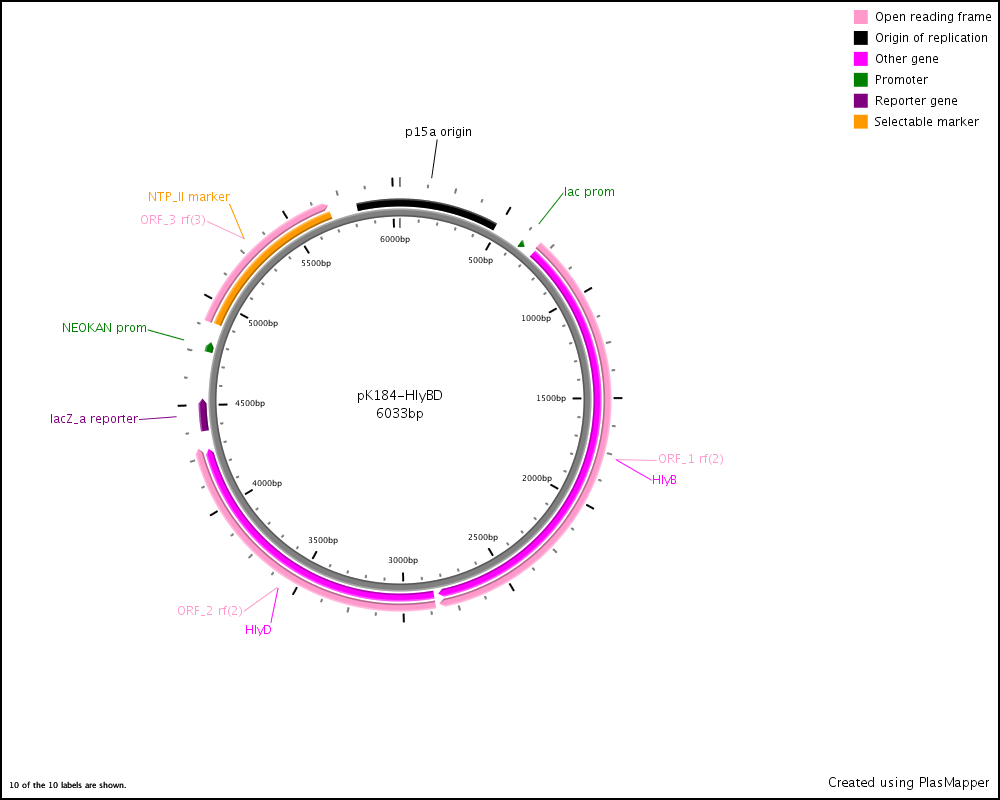


Supplementary Figure 2


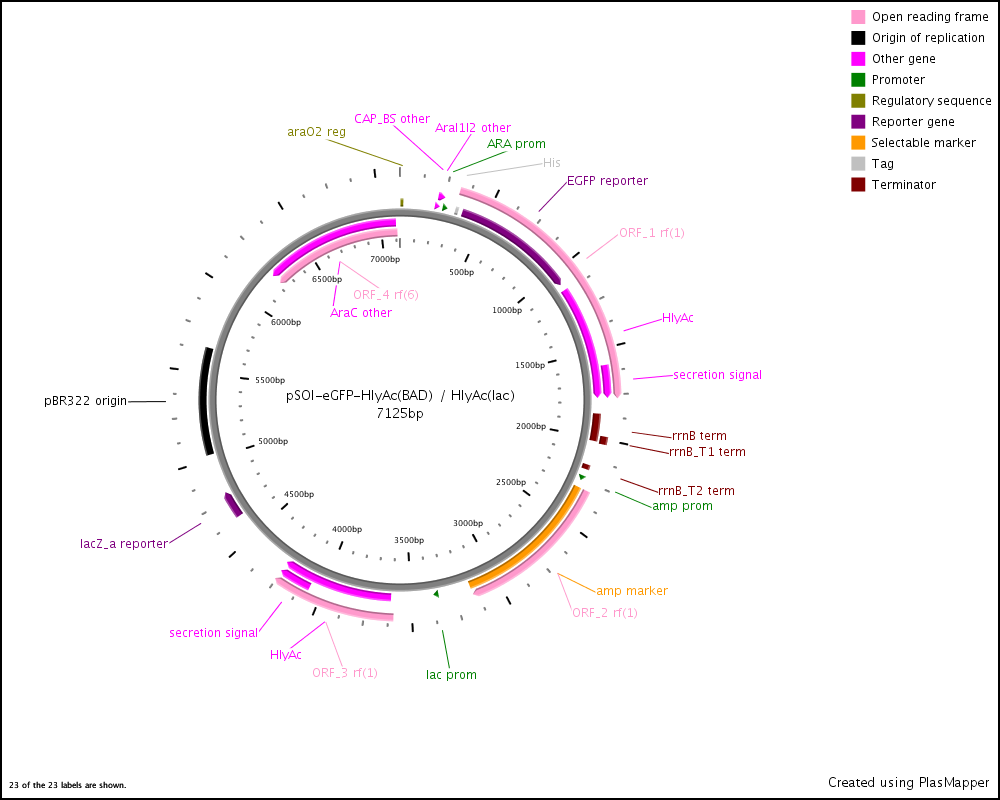


Supplementary Figure 3


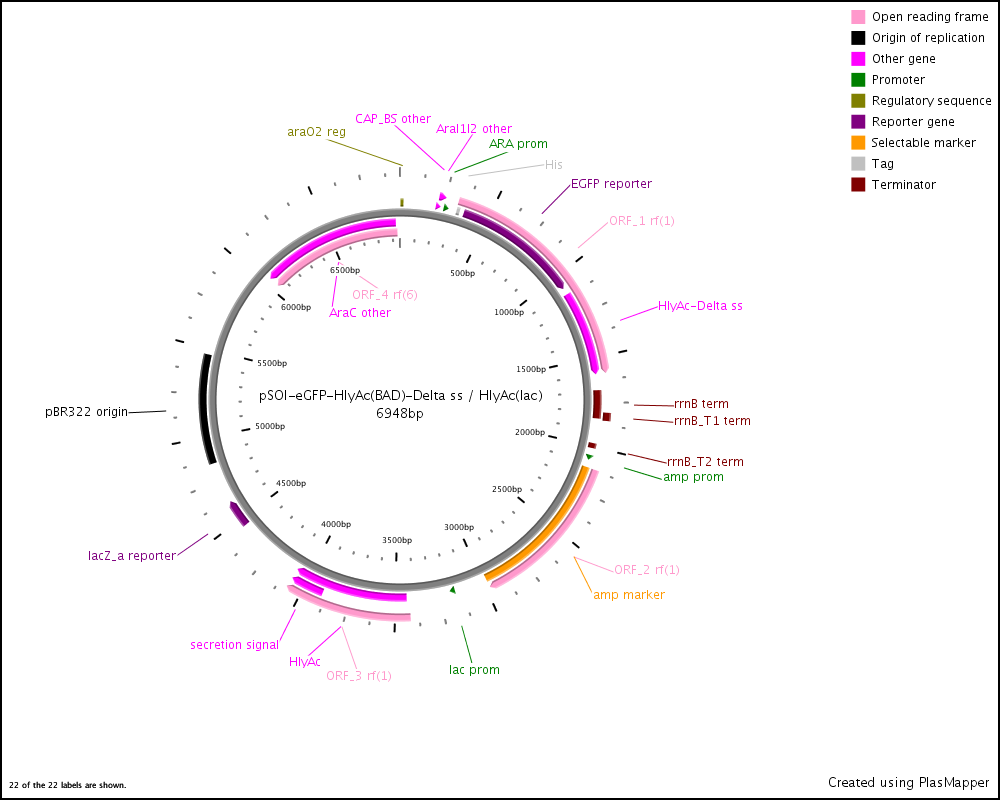


Supplementary Figure 4


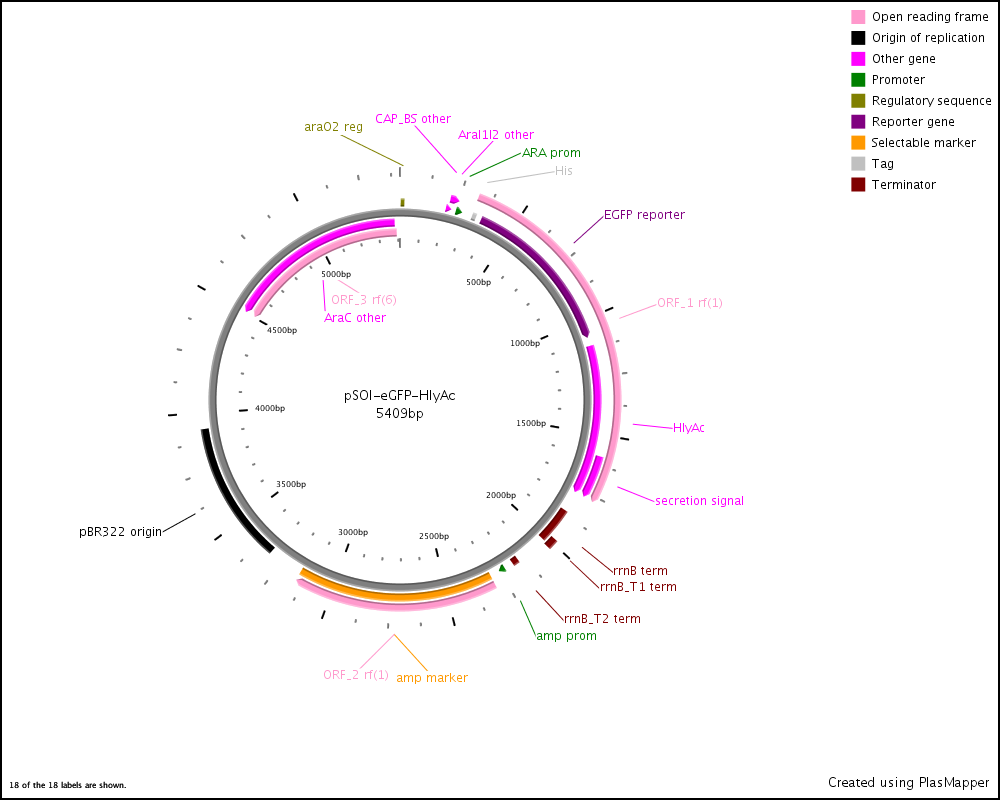


Supplementary Figure 5


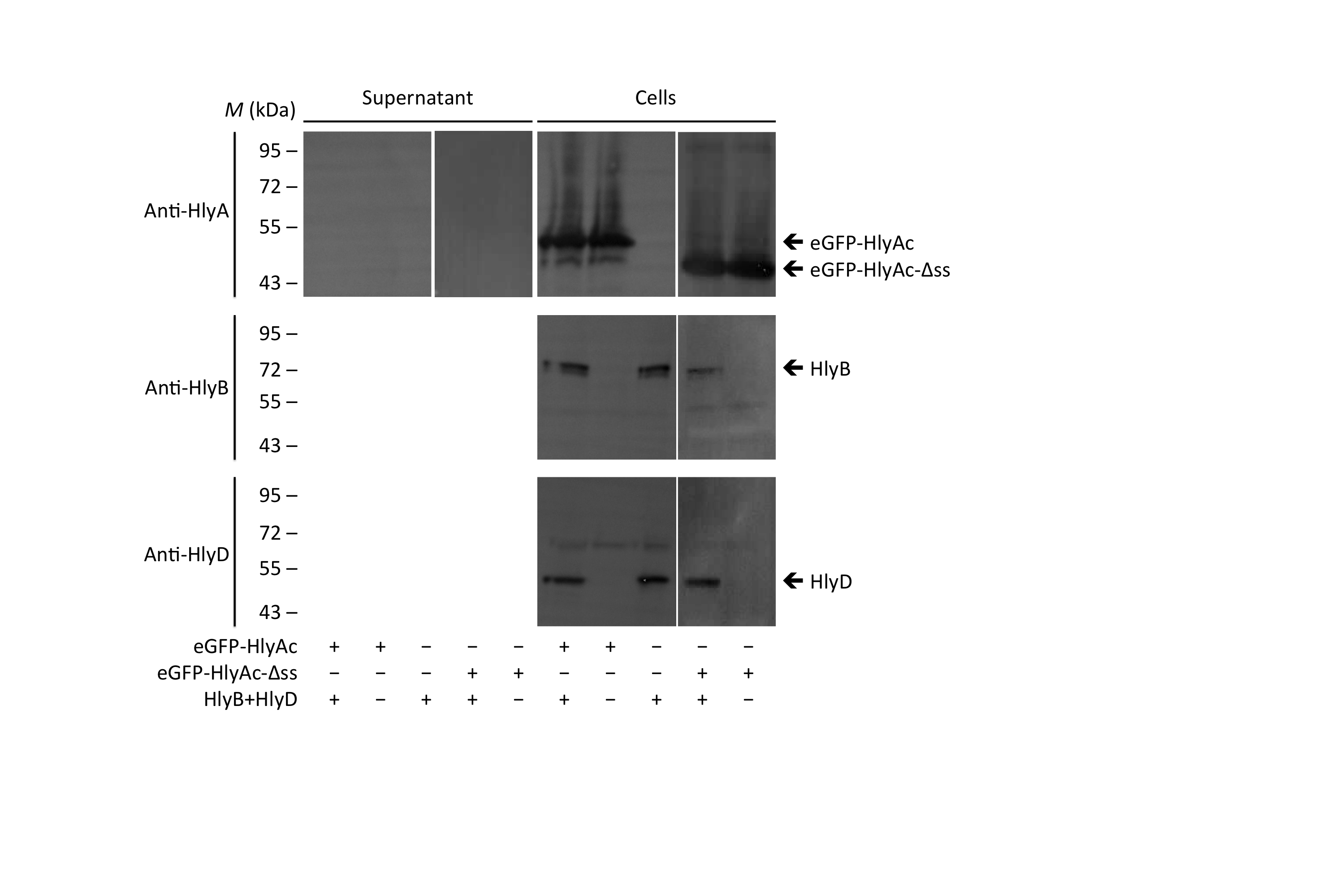


Supplementary Figure 6


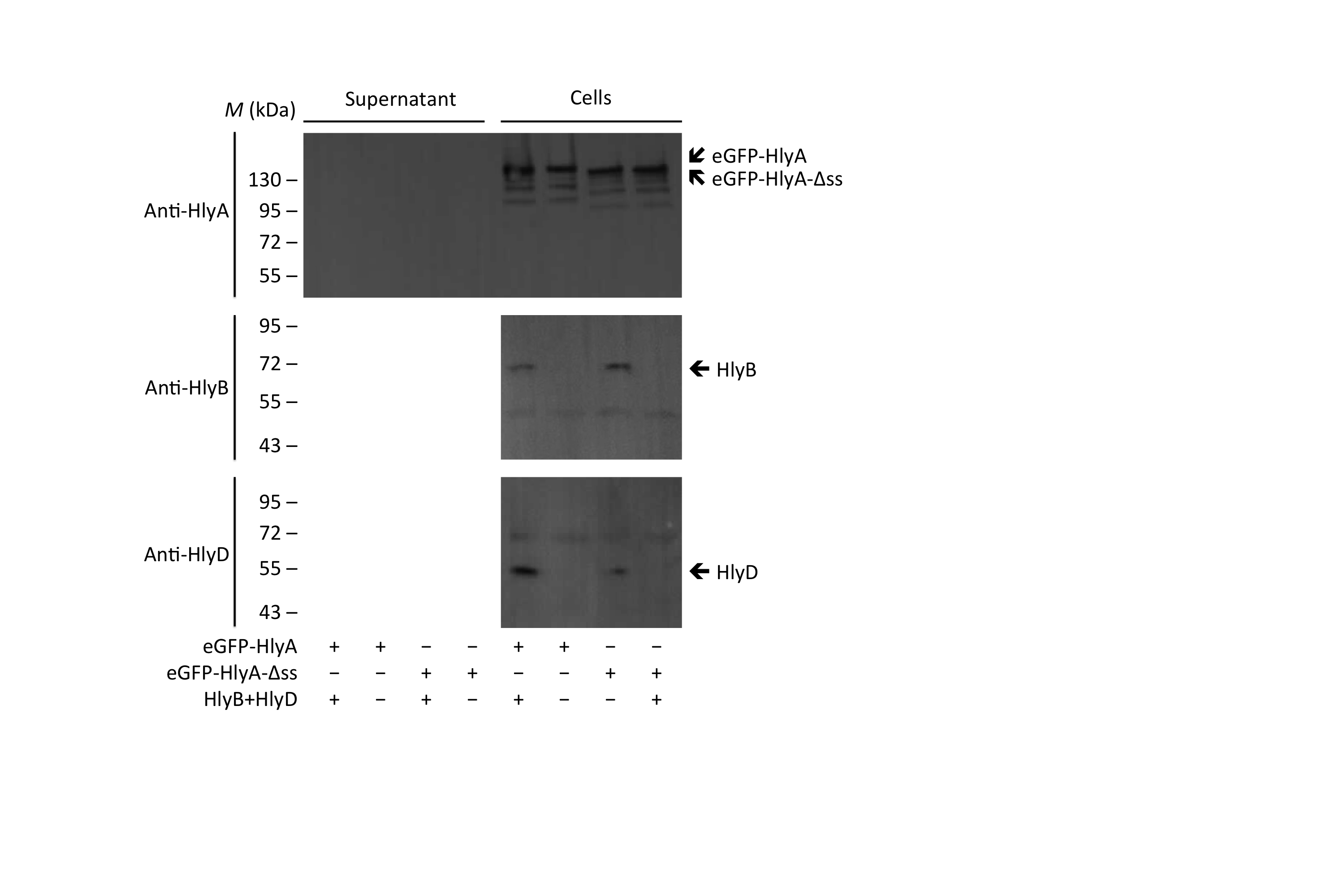


Supplementary Figure 7

Tables

Supplementary Table 1

Primers used in this study

| Name | Sequence |
| --- | --- |
| HlyAcΔ-ss-for | 5’-GGACATGATGCATGAACTTATGGGAG-3’ |
| HlyAcΔ-ss-rev | 5’-CTCCCATAAGTTCATGCATCATGTCC-3’ |
| pSOI-ColE1-for | 5’-CATTTTTAATTTAAAAGGATCTAGGTGAAG-3’ |
| pSOI-AMP-rev | 5’-AGTTTTAAATCAATCTAAAGTATATATGAGTAAAC-3’ |
| Inf-pSOI-HlyA-F | 5’-GATTGATTTAAAACTGCCAATACGCAAACCGCCTCTC-3’ |
| Inf-pSOI-HlyA-R | 5’-TTTAAATTAAAAATGTAGGGGTTCCGCGCACATTTCC-3’ |
| RF_pSOI_eGFP_for | 5’-CCATCATGGTGAGAATTTATATTTTCAAGGTGTGAGCAAGGGCGAGG-3’ |
| RF_pSOI_eGFP_rev | 5’-TGGAAGGGTGGGATTTACCGGACTTGTACAGCTCGTCCATGC-3’ |
| RF_pSOI_HlyA_for | 5’-CCCTTCCAGCATCGAAGGCCGCATGACAACAATAACCACTGCAC-3’ |
| RF_pSOI_HlyA_rev | 5’-TCCGCCAAAACAGCCAAGCTTATGCTGATGTGGTCAGGGT-3’ |
| HlyAΔss_for | 5’-GGGAATGATGCATAAGCCTATGGAAG-3’ |
| HlyAΔss_rev | 5’-CTTCCATAGGCTTATGCATCATTCCC-3’ |
| Deletion-HlyAc-for | 5’-TAAGCTTGGCTGTTTTGGCGGATG-3’ |
| Deletion-HlyAc-rev | 5’-TCATGCATCATGTCCATACACATAACTTACCTT-3’ |

Supplementary Table 2

Plasmids used in this study

| Name | Description | Reference |
| --- | --- | --- |
| pK184-HlyB  pSU-*hlyA*  pSOI-eGFP-HlyAc  pSOI-eGFP-HlyAc-Δss  pSOI-eGFP-HlyAc^BAD^ / HlyAc^lac^  pSOI-eGFP-HlyAc-Δss ^BAD^ / HlyAc^lac^  pSOI-eGFP-HlyA  pSOI-eGFP-HlyA-Δss | Plasmid encoding *hlyB* and *hlyD*  Plasmid *hlyA*  eGFP inserted in pSOI-HlyAc [^2^](#_ENREF_2) *via* restriction free cloning  Plasmid pSOI-eGFP-HlyAc with a stop codon in front of the HlyAc secretion signal *via* site-directed mutagenesis  HlyAc with *lac* promoter inserted in pSOI-eGFP-HlyAc *via* In-Fusion® Advantage PCR Cloning Kit (Clontech)  Plasmid pSOI-eGFP-HlyAc^BAD^ / HlyAc^lac^  without the base pairs coding for the last 60 C-terminal amino acids (HlyAc secretion signal)  HlyAc is exchanged for HlyA from pSU-*hlyA* in plasmid pSOI-eGFP-HlyAc  Plasmid pSOI-eGFP-HlyA with a stop codon in front of the HlyA secretion signal *via* site-directed mutagenesis | [^2^](#_ENREF_2)  [^3^](#_ENREF_3)  This study  This study  This study  This study  This study  This study |

1 Dong, X., Stothard, P., Forsythe, I. J. & Wishart, D. S. PlasMapper: a web server for drawing and auto-annotating plasmid maps. *Nucleic acids research* **32**, W660-664, doi:10.1093/nar/gkh410 (2004).

2 Bakkes, P. J., Jenewein, S., Smits, S. H., Holland, I. B. & Schmitt, L. The rate of folding dictates substrate secretion by the Escherichia coli hemolysin type 1 secretion system. *The Journal of biological chemistry* **285**, 40573-40580, doi:10.1074/jbc.M110.173658 (2010).

3 Thomas, S., Smits, S. H. & Schmitt, L. A simple in vitro acylation assay based on optimized HlyA and HlyC purification. *Analytical biochemistry*, doi:10.1016/j.ab.2014.07.001 (2014).
